# On the Limits of Benzophenone as Cross-Linker for Surface-Attached Polymer Hydrogels

**DOI:** 10.3390/polym9120686

**Published:** 2017-12-07

**Authors:** Esther K. Riga, Julia S. Saar, Roman Erath, Michelle Hechenbichler, Karen Lienkamp

**Affiliations:** Freiburg Center für Interactive Materials and Bioinspired Technologies (FIT) and Department of Microsystems Engineering (IMTEK), Albert-Ludwigs-Universität, Georges-Köhler-Allee 105, 79110 Freiburg, Germany; esther.riga@imtek.uni-freiburg.de (E.K.R.); julia.saar@imtek.uni-freiburg.de (J.S.S.)

**Keywords:** benzophenone cross-linking, coatings, hydrogels, photo-reactive polymers, ring-opening metathesis polymerization (ROMP), surface-attached polymer networks

## Abstract

The synthesis of different photo-reactive poly(alkenyl norbornenes) and poly(oxonorbornenes) containing benzophenone (BP) via ring-opening metatheses polymerization (ROMP) is described. These polymers are UV irradiated to form well-defined surface-attached polymer networks and hydrogels. The relative propensity of the polymers to cross-link is evaluated by studying their gel content and its dependency on BP content, irradiation wavelength (254 or 365 nm) and energy dose applied (up to 11 J·cm^−2^). Analysis of the UV spectra of the polymer networks demonstrates that the poly(oxonorbornenes) show the expected BP-induced crosslinking behavior at 365 nm, although high irradiation energy doses and BP content are needed. However, these polymers undergo chain scission at 254 nm. The poly(alkenyl norbornenes), on the other hand, do not cross-link at 365 nm, whereas moderate to good cross-linking is observed at 254 nm. UV spectra demonstrate that the cross-linking at 254 nm is due to BP cross-linking combined with a [2 + 2] cylcoaddition of the alkenyl double bonds. This indicates limitations of benzophenone as a universally applicable cross-linking for polymer networks and hydrogels.

## 1. Introduction

**Figure 1:**
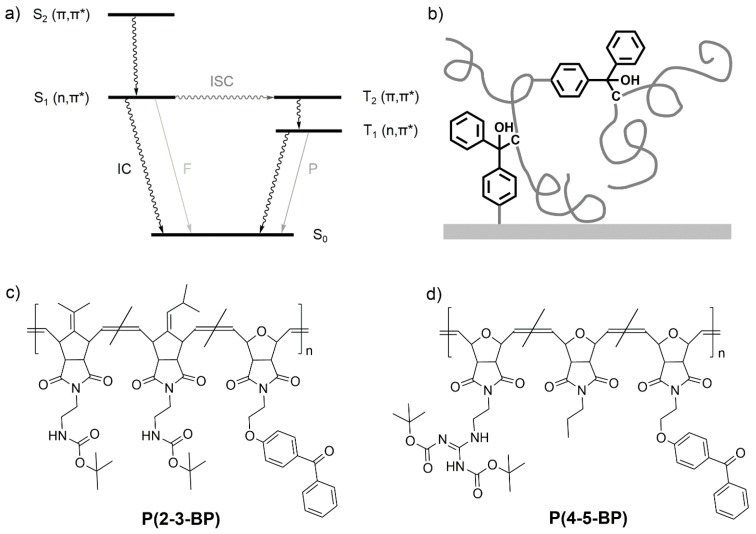
(**a**) Simplified Jablonski diagram of benzophenone illustrating its electronic states and possible transitions by internal conversion (IC), intersystem crossing (ISC), fluorescence (F) and phosphorescence (P) [16]; (**b**) Cartoon illustration of a surface-attached polymer network with benzophenone as surface anchor group and chain-chain cross-linker; (**c**,**d**) Structure of the poly(alkenylnorbornenes) **P(2-3-BP)** and poly(oxonorbornenes) **P(4-5-BP)** used in this study.

Benzophenone (BP) is well-known for its photo-reactivity, which has been extensively reviewed [1,2]. In particular, BP forms covalent C–C bonds to molecules containing C–H bonds by UV-activated C, H–insertion reactions. More precisely, BP can absorb a photon upon UV irradiation at λ ~ 350 nm. This causes an electronic transition from the nonbonding *n*-orbital of oxygen to the antibonding π*-orbital of the carbonyl group (Figure 1a). Slightly more energy (i.e., irradiation at λ ~ 250 nm) is needed to induce the transition of an electron from the bonding π-orbital to the antibonding π*-orbital of the carbonyl group (Figure 1a). Either way, the singlet diradicaloid thus formed undergoes intersystem crossing (ISC, Figure 1) into a triplet state, so that a diradical with one unpaired electron centered on oxygen and another one on the carbon of the former carbonyl group is generated. The oxygen can now interact with C–H σ-bonds of neighboring molecules (in our case polymer chains) by abstracting a hydrogen atom, thus forming an OH bond. This creates a radical on the carbon atom left behind. The new C radical can combine with the C radical on the former BP carbonyl C atom, so that a covalent C–C bond between the former BP group and the polymer chain is formed (Figure 1b) [1,3]. The excitation of benzophenone is reversible, i.e. the diradical formed falls back into the ground state if no suitable reaction partner is found; thus its excitation can be repeated until favorable conditions (e.g., suitable reaction geometry and distance) for the reaction with a neighboring C–H-group are obtained [1]. As a result, efficient covalent modifications can be obtained using benzophenone [1]. Benzophenone was initially used in biochemistry (e.g., as labeling reagent for photo-affinity probes) [4,5,6,7,8], since the longer wavelengths needed for BP activation (λ ~ 360 nm) do not damage sensitive materials including proteins [9]. Prucker et al. showed that BP could be used as anchor group to functionalize surfaces with thin polymer layers [10]. Based on this principle, polymer monolayers made from poly(vinyl alcohol), poly(styrene), poly(acrylic acid) and poly(methacrylic acid) have been attached to surfaces [10,11]. Next, BP-containing polymers that can self-crosslink upon UV irradiation were developed, so that surface-attached polymer networks and hydrogels based on BP could be obtained [12,13,14]. Besides being a versatile reagent for covalent cross-linking, benzophenone can also be used but as a photo-initiator to induce cross-linking [15].

In spite of its overall versatility, there are some limitations to the use of BP in polymer chemistry. For example, Rupp et al. observed chain scission when attempting to cross-link polyethylene oxide (PEO) in ionic liquids using benzophenone as a photo-initiator [17]. Teixeira et al. found that PEO would undergo chain scission at low concentrations of the added BP photo-initiator, while at higher BP concentrations cross-linking became dominant [18]. Christensen et al. analyzed the UV-induced gelation of acrylate copolymers containing covalently attached benzophenone [19]. They found that when highly abstractable hydrogen atoms were present on the main polymer chain, BP induced chain cleavage rather than cross-linking [19]. 

In our work on surface-attached polymer networks and hydrogels made from poly(norbornenes), we came across two other BP-containing polymer families that failed to show the anticipated cross-linking behavior. The aim of this work was therefore to systematically study UV-activated BP cross-linking of these polymers, and to identify structural parameters of the polymers that prevented cross-linking. For this, we synthesized various BP-containing poly(alkenylnorbornenes) and poly(oxo-norbornenes) (Figure 1c,d, respectively) via ring-opening metathesis polymerization (ROMP). These consisted of alkenylnorbornene imide and oxonorbornene imide repeat units, and contained a quite large number of heteroatoms and double bonds. The imide sites were substituted either with BP moieties, propyl groups, or *N*-Boc protected amine/guanidin groups. By comparing the cross-linking behavior of these polymers when UV irradiated at λ = 254 or 365 nm, we could identify polymer components that are unfavorable for polymer cross-linking using benzophenone. Thus, we could derive design rules for the next generation of benzophenone cross-linkable poly(norbornenes), and potentially also for (surface-attached) networks and hydrogels made from other polymers.

## 2. Experimental

### 2.1. Materials

All chemicals were obtained as reagent grade from Sigma-Aldrich (Taufkirchen, Germany), Carl Roth (Karlsruhe, Germany), Fluka (Taufkirchen, Germany), or Alfa Aeser (Karlsruhe, Germany), and used as received. High performance liquid chromatography (HPLC) grade solvents were purchased dry from Carl Roth (Karlsruhe, Germany), and used as received. Dichloromethane (DCM) was freshly distilled over P_2_O_5_ before use.

### 2.2. Instrumentation

Gel permeation chromatography (GPC, in chloroform, calibrated with poly(methyl methacrylate) standards) was performed on a PSS SDV column (PSS, Mainz, Germany). NMR spectra were recorded on a Bruker 250 MHz spectrometer (Bruker, Madison, WI, USA) using acetone, MeOD and CDCl_3_ as solvent and tetramethylsilane (TMS) as internal reference. The thickness of the dry polymer layers on silicon wafers was measured with the auto-nulling imaging ellipsometer Nanofilm EP3 (Nanofilm Technologie GmbH, Göttingen, Germany), which was equipped with a 532 nm solid-state laser. For each sample, the average value from three different positions was taken. The irradiation of samples with UV light was conducted using a BIO-LINK Box (Vilber Lourmat GmbH, Eberhardzell, Germany) with different wavelengths (254 and 365 nm). UV spectra were taken using a Varian Cary 50 system (Varian, Darmstadt, Germany).

### 2.3. Synthesis

#### 2.3.1. Synthesis of the Benzophenone-Containing Monomer **M1**

**Scheme 1:**
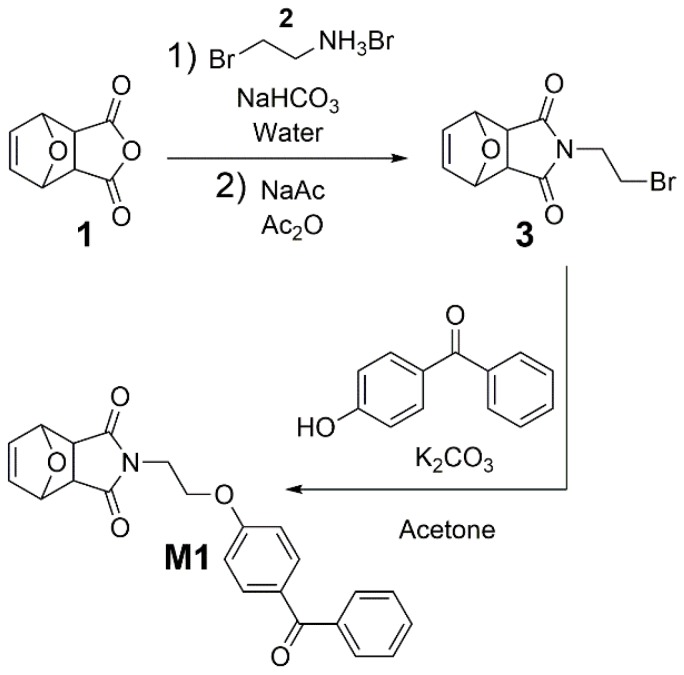
Synthesis of the benzophenone-containing monomer **M1**. The oxonorbornene anhydride **1** is reacted with the brominated amine **2** to form the oxonorbornene imide **3**. The benzophenone group is introduced in the second reaction step to yield the monomer **M1**.

The benzophenone-containing monomer (**M1**) shown in Scheme 1 was synthesized under nitrogen using standard Schlenk techniques. Oxonorbornene (**1**) was reacted with the brominated amine **2** following a literature procedure to yield *N*-(2-bromoethyl)-3,6-tetrahydrophthalimide (3) [20]. In the second step, 4-hydroxybenzophenone (19.8 g, 100 mmol) and K_2_CO_3_ (13.8 g, 100 mmol) were dissolved in dry acetone (250 mL), and the reaction mixture was stirred at 60 °C. Oxonorbornene imide **3** (2.72 g, 10.0 mmol) was slowly added, and the reaction mixture was refluxed for 24 h. For work up, water was added and the solution was extracted with DCM. The combined organic phases were washed with NaOH solution (10%) and dried with MgSO_4_. The solvent was evaporated under reduced pressure. The thus obtained product was dissolved in DCM and hexane was added slowly, resulting in two solvent layers. The vial was put in the freezer allowing a slow mixing of the solvents, resulting in the crystallization of the pure product. After removing the solvent, the isolated yield was about 38% (1.48 g). ^1^H NMR (250 MHz, acetone, δ): 7.44–7.89 (m, 7H, benzophenone-CH), 7.04 (d, 2H, ^2^J_H–H_ = 8.85, benzophenone-CH), 6.58 (s, 2H, C=CH), 5.16 (s, 2H, C=CH–CH), 4.25 (t, 2H, ^3^J_H–H_ = 5.84, N–CH_2_–CH_2_), 3.88 (t, 2H, ^3^J_H–H_ = 5.80, N–CH_2_–CH_2_), 2.96 (s, 2H, CH–C=O); ^13^C NMR (63 MHz, acetone, δ): 195.25, 177.05, 163.10, 139.32, 137.47, 133.09, 132.79, 131.36, 130.33, 129.22, 115.23, 81.85, 65.23, 48.42, 38.42, 29.92; FTMS (ESI) *m*/*z*: (M + H)^+^ calcd for C_23_H_20_O_5_N, 390.13; found, 390.13.

#### 2.3.2. Synthesis of Model Polymer **P(2-3-BP)**

**Scheme 2:**
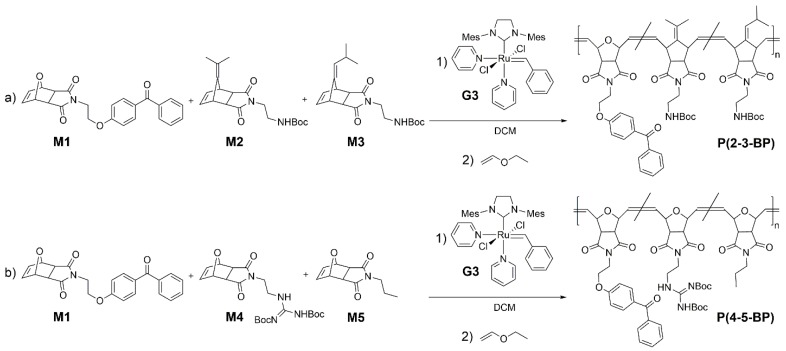
Copolymerization of **M1** with (**a**) norbornene derivatives **M2** and **M3**, and (**b**) oxonorbornene imides **M4** and **M5** via ring-opening metathesis polymerization. The monomers were initiated with Grubbs’ third generation catalyst **G3**. The living chain end was then quenched with ethyl vinyl ether to yield the terpolymers **P(2-3-BP)** and **P(4-5-BP)**, respectively.

To obtain **P(2-3-BP)**, monomer **M1** was copolymerized with the alkyl-functionalized norbornene monomers **M2** and **M3** (Scheme 2), which were synthesized following the method reported by Ilker [21]. Grubbs’ third generation catalyst was synthesized as described by Love [22]. The amount of each reagent used, and the molecular weight of the resulting polymers, as determined by GPC, can be found in the supporting information (Table S1). A typical polymerization was performed under nitrogen atmosphere using standard Schlenk techniques. Monomers **M2** and/or **M3** were dissolved in dry DCM (5 mL) and stirred for 30 min. Grubbs’ third generation catalyst **G3** was dissolved in dry DCM (1 mg·mL^−1^) in a second flask and added to the monomer solution. The reaction mixture was allowed to react for 2 h at 40 °C, and a solution of **M1** in dry DCM (10 mL) was added dropwise simultaneously. Subsequently, an excess of ethylvinyl ether (1 mL) was added to terminate the reaction. After precipitation in ice-cold hexane (300 mL), the product was dried under reduced pressure. The isolated yields were between 18% and 67%. ^1^H NMR (250 MHz, CDCl_3_, δ): 6.85–7.85 (*m*, 9H, benzophenone-CH), 4.85–5.70 (*m*, 2H, C=CH), 3.50–3.83 (*m*, 4H, CH_2_ and N–CH_2_), 3.00–3.40 (*m*, 4H, C=C–CH and CH), 2.20–2.35 (*m*, 1H, M3–CH_3_–CH), 1.57–1.75 (*m*, 6H, M2–CH_3_), 1.41 (*s*, 9H, boc-CH_3_), 0.79–0.90 (*m*, 6H, M3–CH_3_); ^13^C NMR (63 MHz, CDCl_3_, δ): 179.28, 177.63, 163.18, 161.45, 156.02, 142.51, 137.91, 137.67, 137.21, 120.59, 104.21, 97.26, 93.73, 84.31, 79.48, 79.29, 54.38, 48.90, 47.91, 45.64, 38.53, 29.68, 28.34, 28.05, 26.26, 23.09, 21.57, 19.56.

For cleavage of the tert-butyl carbamate group (*N*-Boc protective group), the polymer made from 2.5% **M1**, 65% **M2** and 32.5% **M3** (60 mg, 0.85 mmol) was dissolved in trifluoroacetic acid (2 mL) and allowed to react for 20 h at 45 °C. The crude product was precipitated in DCM (200 mL) and dried under reduced pressure. The isolated yield was roughly 30% (20 mg). ^1^H NMR (250 MHz, MeOD-d4, δ): 6.86–7.84 (*m*, 9H, benzophenone-CH), 5.53–5.71 (*m*, 2H, C=CH), 3.67–3.90 (*m*, 4H, CH_2_ and N–CH_2_), 3.05–3.37 (*m*, 4H, C=C–CH and CH), 2.13–2.27 (*m*, 33% 1H, M3–CH_3_–CH), 1.61–1.80 (*m*, 66% 6H, M2–CH_3_), 0.85–1.00 (*m*, 33% 6H, M3–CH_3_).

#### 2.3.3. Synthesis of Model Polymer **P(4-5-BP)**

To obtain **P(4-5-BP)**, monomer **M1** was copolymerized with the oxonorbornene monomers **M4** and **M5** shown in Scheme 2, which were synthesized following literature procedures [23,24]. The amount of each reagent used, and the molecular weight of the resulting polymer, as determined by GPC, can be found in the supporting information (Table S4). A typical polymerization was performed under nitrogen atmosphere using standard Schlenk techniques. Monomers **M1**, **M4** and/or **M5** were dissolved in dry DCM (3 mL) and stirred for 30 min. Grubbs’ third generation catalyst was dissolved in dry DCM (1 mg·mL^−1^) in a second flask and added to the monomer solution. The reaction mixture was allowed to react for 30 min at room temperature. Subsequently, an excess of ethylvinyl ether (0.6 mL) was added to terminate the reaction. After precipitation in ice-cold hexane (300 mL), the product was dried under reduced pressure. The isolated yields were about 90%. ^1^H NMR (250 MHz, CDCl_3_, δ): 6.89–7.85 (*m*, 9H, benzophenone-CH), 6.02–6.10 (*m*, 1H, C=CH, *trans*), 5.70–5.83 (*m*, 1H, C=CH, *cis*), 4.93–5.15 (*m*, 1H, C=C–CH, *cis*), 4.40–4.55 (*m*, 1H, C=C–CH, *trans*), 3.60–3.80 (*m*, 4H, CH_2_ and N–CH_2_), 3.27–3.47 (*m*, 2H, CH), 1.63–1.80 (*m*, 2H, M5–CH_2_), 1.48 (*s*, 18H, boc-CH_3_), 0.81–0.90 (*m*, 3H, M5–CH_3_).

#### 2.3.4. Synthesis of Surface-Attached Polymer Networks

Silicon substrates functionalized with benzophenon were prepared as follows: First, the cross-linking agent triethoxy benzophenone silane (3EBP-silane) was synthesized as described in the literature [25]. A solution of 3EBP-silane (40 mmol·L^−1^ in toluene) was then spin coated on a standard Si wafer (800 rpm, 120 s and 500 rpm s^−1^). Subsequently, the wafer was cured for 30 min at 100 °C on a preheated hot plate, washed with organic solvents (first with toluene to remove unbound **3EBP**-silane, then ethanol for removal of the polar leaving groups and easier surface drying). The surfaces were then dried under a continuous flow of nitrogen gas.

To synthesize surface-attached polymer networks from **P(2-3-PB)** or **P(4-5-BP)**, a solution of the respective polymer in chloroform (20 mg·mL^−1^) was spin cast on a 3-EBP treated silicon wafer (3000 rpm, 30 s and 1000 rpm·s^−1^). The films were cross-linked at 254 or at 365 nm with different energy doses, washed with DCM to remove unattached polymer chains, and dried in a nitrogen stream. For deprotection, the samples were deposited in 4 molar hydrochloric acid in dioxane for 12 h, washed with ethanol and dried in a nitrogen stream.

## 3. Results and Discussion

The benzophenone-containing poly(alkenylnorbornenes) **P(2-3-BP)** (Figure 1c) and poly(oxonorbornenes) **P(4-5-BP)** (Figure 1d) used in this study were obtained by copolymerizing the benzophenone-containing monomer **M1** (Scheme 1) with the respective functional monomers **M2** to **M5** (Scheme 2). To obtain **M1**, first oxonorbornene anhydride **1** was reacted with the brominated amine **2** to give *N*-(2-bromoethyl)-3,6-tetrahydrophthalimide (**3**, Scheme 1). The latter was then reacted with hydroxybenzophenone and K_2_CO_3_ in dry acetone for 24 h under reflux, yielding monomer **M1** (Scheme 1). Details are given in the Experimental. 

To synthesize different variants of the target poly(alkenylnorbornene) **P(2-3-BP)**, monomer **M1** and the alkenylnorbornene derivatives **M2** and **M3** were mixed in various monomer feed ratios and polymerized via ring opening metathesis polymerization (ROMP) using the Grubbs’ third generation catalyst (**G3**) (Scheme 2a). The catalyst-to-monomer ratio was adjusted to obtain copolymers with the desired molecular weight (target molecular weight 100,000 g·mol^−1^). The target content of BP repeat units was 2.5% to 5%. Since the polymerization rates of the monomers used varied significantly (**M2** and **M3** being much less reactive than the oxonorbornene imide **M1**) [26,27], the BP-containing monomer **M1** was added dropwise over 120 min to the reaction mixture containing **M2**, **M3** and the polymerization catalyst. That way, a more homogeneous distribution of the BP-monomer units along the polymer chain was obtained. The molar composition of the polymers thus obtained, and their molar mass distribution parameters (determined by gel permeation chromatography) are summarized in Table 1. It was confirmed by ^1^H NMR spectroscopy via a comparison of the integrated peaks of the benzophenone group (6.85 to 7.85 ppm, 9H) and the *N*-Boc protective group (1.41 ppm, 9H) that the actual BP content of the polymers matched the target content.

To obtain different variants of the polymers **P(4-5-BP)**, monomer **M1** was copolymerized with different amounts of the oxonorbornene derivatives **M4** and **M5** (Scheme 2b). These three monomers had similar polymerizable groups and therefore similar polymerization rates, and were thus mixed and added at the beginning of the reaction. The target BP content was 5 to 15 mol %. As before, the comonomer feed ratio and catalyst-to-monomer ratio were adjusted to obtain the desired copolymer molecular weights and compositions (Table 1). For some of the network formation studies presented below, the *N*-*tert*-butyloxycarbonyl (*N*-Boc) protective groups of the polymers were cleaved with trifluoroacetic acid before cross-linking. Unless otherwise specified, the reactions and measurements were performed on the protected polymers.

To synthesize surface-attached polymer networks from the **P(4-5-BP**) polymers, they were dissolved in dichloromethane (DCM) and spin-coated onto silicon wafers that had been previously functionalized with benzophenone using the anchor group triethoxy benzophenone silane (**3EBP**) [25]. **3EBP** contains a benzophenone moiety and a silane group, which links it covalently to the silicon surface. Upon UV irradiation, the BP moiety of the **3EBP** anchor group formed a covalent bond to **P(4-5-BP**) and thus attached it to the surface, while the BP moieties that were part of **P(4-5-BP)** simultaneously formed cross-links between the polymer chains, so that overall a surface-attached polymer network was obtained (Figure 1b).

The degree of cross-linking of a network or hydrogel can be quantified by determining its gel content. The gel content of the here presented polymer films was determined by first measuring the overall dry film thickness *d*_0_ of the networks just after UV irradiation via ellipsometry (i.e., the thickness of the gel plus the sol). The networks were then extracted in DCM overnight to remove unbound polymer chains. After this treatment, the remaining dry layer thickness *d*_extract_ (i.e., the gel thickness) was also measured via ellipsometry. From this, the gel content of each layer was calculated using Equation (1).

(1) $$\mathrm{gel}\;\mathrm{content}=(\frac{d_{\mathrm{extract}}}{d_{0}}\times 100)\;\%$$

**Figure 2:**
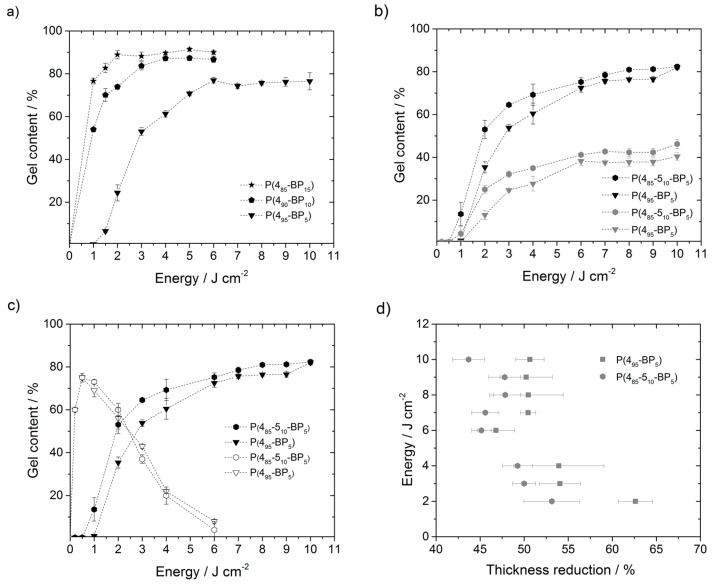
Gel content of surface-attached polymer networks and hydrogels made from different **P(4-5-BP)** polymers as a function of the irradiation energy dose: (**a**) Polymers with varying amount of benzophenone repeat units (5–15 mol %, respectively), irradiated at λ = 365 nm (Table S4); (**b**) polymers with 5% benzophenone repeat units and varying amounts of co-repeat units, black symbols = *N*-Boc protected polymers, grey symbols = deprotected polymers, irradiated at λ = 365 nm (Table S5); (**c**) Comparison of cross-linking of the protected **P(4_85_-5_10_-BP_5_)** and **P(4_95_-BP_5_)** at λ = 365 nm (filled symbols, Table S5) and λ = 254 nm (open symbols, Table S3); (**d**) Thickness reduction due to deprotection of the *N*-Boc protected polymeric layers consisting of **P(4_85_-5_10_-BP_5_)** and **P(4_95_-BP_5_)** as a function of the irradiation energy dose (Table S5). **P(4_95_-BP_5_) =** (**M1**/**M4**/**M5** 5:95:0), **P(4_90_-BP_10_)** = (**M1**/**M4**/**M5** 10:90:0), **P(4_85_-BP_15_)** (**M1**/**M4**/**M5** 15:85:0)) (**P(4_85_-5_10_-BP_5_)** (**M1**/**M4**/**M5** 5:85:10) and **P(4_95_-BP_5_)** (**M1**/**M4**/**M5** 5:95:0).

When different polymers are cross-linked using comparable conditions (e.g., the same irradiation doses and wavelengths), the gel content of the networks or hydrogels formed can be considered as a quantitative measure for the relative propensity of each polymer to cross-link. With this in mind, the gel content of networks made from different members of the **P(2-3-BP)** and the **P(4-5-BP)** families was determined after UV irradiation at different wavelengths (λ = 254 or 365 nm, respectively) and different energy doses. In Figure 2, the gel content of the **P(4-5-BP)** polymer networks thus obtained is shown. (For these polymers, the subscripts indicate the relative amount of each repeat unit, i.e., **P(4_85_-5_10_-BP_5_)** refers to a polymer made from 85% **M4**, 10% **M5**, and 5% **M1**, and **P(4_95_-BP_5_)** to a polymer made from 95% **M4**, 0% **M5**, and 5% **M1**.) The thickness data onto which the gel content curves are based are listed in the Supporting Information (Tables S3–S5).

Figure 2a shows the gel content of **P(4-BP)** networks with 5–15 mol % of BP for various irradiation energy doses at λ = 365 nm. In this graph, the gel content first increases steeply to the optimum dose, and then levels off into a plateau at higher energy doses. This is the usual behavior described for BP cross-linked polymers in the literature [15], even though the energy doses needed to obtain a substantial amount of cross-linking in this polymer were quite high. Also in line with theory [12], the data shows that layers made from polymers with a higher BP content also had a higher gel content when the same irradiation energy dose was used. For example, **P(4_85_-BP_15_)** irradiated with 2 J·cm^−2^ had a gel content of around 90%, while the gel content of **P(4_90_-BP_10_)** was only 74%, and that of **P(4_95_-BP_5_)** was only 24%. It is noteworthy, though, that surprisingly high BP contents and energy doses were needed to obtain significant amounts of cross-linking.

When the **P(4-5-BP)** polymer networks were treated with acid to remove their *tert*-butyloxycarbonyl (Boc) protective groups (to obtain the corresponding polymer hydrogels), a layer thickness decrease of 46% to 63% was observed. For example, the layer thickness of **P(4_95_-BP_5_)** (energy dose 10 J·cm^−2^) decreased from 229 to 113 nm after deprotection. This was mostly caused by the high mass loss occurring when the Boc groups were removed (42 mass %). At the same time, though, some polymer chains that are cross-linked predominantly via bonds to these Boc groups might also become detached, which would further increase the mass loss. To investigate if such chains formed was a significant fraction of the polymer networks obtained, the gel content of networks made from protected and the deprotected polymers **P(4_95_-BP_5_)** (irradiated with different energy doses at λ = 365 nm) were determined and compared to the gel contents of the corresponding **P(4_85_-5_10_-BP_5_)** polymers. **P(4_85_-5_10_-BP_5_)** had the same benzophenone content as **P(4_95_-BP_5_)**, but 10 mol % repeat units containing propyl substituents, and thus more C–H bonds available for cross-linking that would not be cleaved by deprotection (Figure 1d). When irradiated at 365 nm, **P(4_85_-5_10_-BP_5_)** consistently had higher gel contents than **P(4_95_-BP_5_)** throughout the energy dose range investigated (Figure 2b), even though it had a lower absolute number of C–H bonds compared to **P(4_95_-BP_5_)**. For example, **P(4_85_-5_10_-BP_5_)** had a gel content of around 81% after illumination with 8 J·cm^−2^, whereas that of **P(4_95_-BP_5_)** was only 76% when irradiated with the same energy dose. This indicates that the propyl C–H groups per se were more easily available for cross-linking than the C–H bonds of the two CH_2_CH_2_N-Boc fragments. The same trend was observed for the corresponding deprotected polymers of **P(4_85_-5_10_-BP_5_)** and **P(4_95_-BP_5_)** (Figure 2b). In Figure 2d, the relative thickness loss due to deprotection (in %) of the two polymers is compared. When all Boc groups are cleaved, the theoretical mass loss of deprotected **P(4_95_-BP_5_)** should be 42 mass %, corresponding to a thickness reduction of roughly the same percentage, while that of **P(4_85_-5_10_-BP_5_)** should be only slightly lower (40 mass %). Figure 2d shows that the mass loss for the deprotected **P(4_95_-BP_5_)** polymers was considerably larger than for the **P(4_85_-5_10_-BP_5_)** polymers, indicating that the former contained a larger fraction of polymer chains that were exclusively cross-linked via bonds to the Boc groups. In line with this interpretation, the data also shows that for both polymers, the thickness decrease becomes smaller with increasing irradiation energy, i.e., in the strongly irradiated polymer networks (and the corresponding deprotected hydrogels), more cross-links to polymer parts other than the Boc group could be formed.

**Figure 3:**
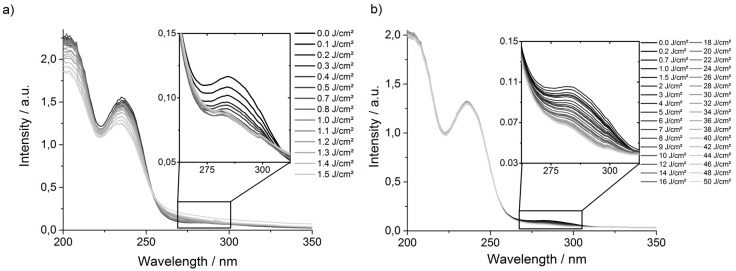
UV absorption spectra of **P(4_95_-BP_5_)** films irradiated at (**a**) λ = 254 nm with 0 to 1.5 J·cm^−2^ and (**b**) λ = 365 nm with 0 to 50 J·cm^−2^. The insets show the reduction of the adsorption band of the benzophenone moieties as a function of the irradiation energy.

To look more closely into the aspect of the surprisingly high-energy doses and cross-linker contents needed for substantial cross-linking of the **P(4-5-BP)** polymers, **P(4_85_-5_10_-BP_5_)** and **P(4_95_-BP_5_)** were both irradiated with higher energy photons (λ = 254 nm) and different energy doses (Figure 2c). Under these conditions, a significant amount of cross-linking was achieved even with an energy dose as low as 0.5 J·cm^−2^ (Figure 2c). For example, polymer **P(4_95_-BP_5_**) had a layer thickness of 211 nm and a gel content of 75% when irradiated with 0.5 J·cm^−2^. However, when this polymer was irradiated with higher doses (up to 6 J·cm^−2^), the layer thickness decreased to 20 nm and the gel content to only 8%. This effect was also observed for the copolymer **P(4_85_-5_10_-BP_5_)** (Figure 2c). This indicates that higher energy photons could, in principle, more efficiently cross-link these polymers. However, when too many photons of that energy were provided, unfavorable side reactions such as polymer main chain scissions, leading to lower gel content, become dominant. To confirm this, UV absorption spectra of **P(4_95_-BP_5_)** films that had been irradiated at 254 nm with different energy doses were measured (Figure 3a).

These spectra showed that the UV absorption band of the BP group (at ~280 nm) decreased continuously when irradiated with energy doses from 0 to 0.5 J·cm^−2^ at λ = 254 nm (Figure 3a), i.e., the BP groups were quantitatively consumed in that energy range. When the samples were exposed to higher energy doses at that wavelength, the whole shape of the curve started to alter, particularly the peaks at 200 and 235 nm decreased significantly. This confirms that other reactions besides BP activation also occurred (Figure 2c). It can be speculated that when the BP groups were used up, the 254 nm photons could interact with other functional groups, which could have led to cleavage of the polymer backbone. When the **P(4_95_-BP_5_)** films were irradiated with less energetic photons (λ = 365 nm), the UV absorption spectra did not show a decrease in the region of 200 to ~260 nm, even for energy doses as high as 50 J·cm^−2^ (Figure 3b). Instead, a steady decrease of the UV absorption band of the BP group at 280 nm with increasing energy dose was observed (Figure 3b). Thus, these lower energy photons had a lower probability to trigger cross-linking via the BP group, and it therefore took higher energy doses to obtain similarly high gel contents as for the 254 nm photons. On the other hand, the 365 nm photons did not UV-activate the other functional groups in the polymer, so that the desired polymer networks were obtained.

**Figure 4:**
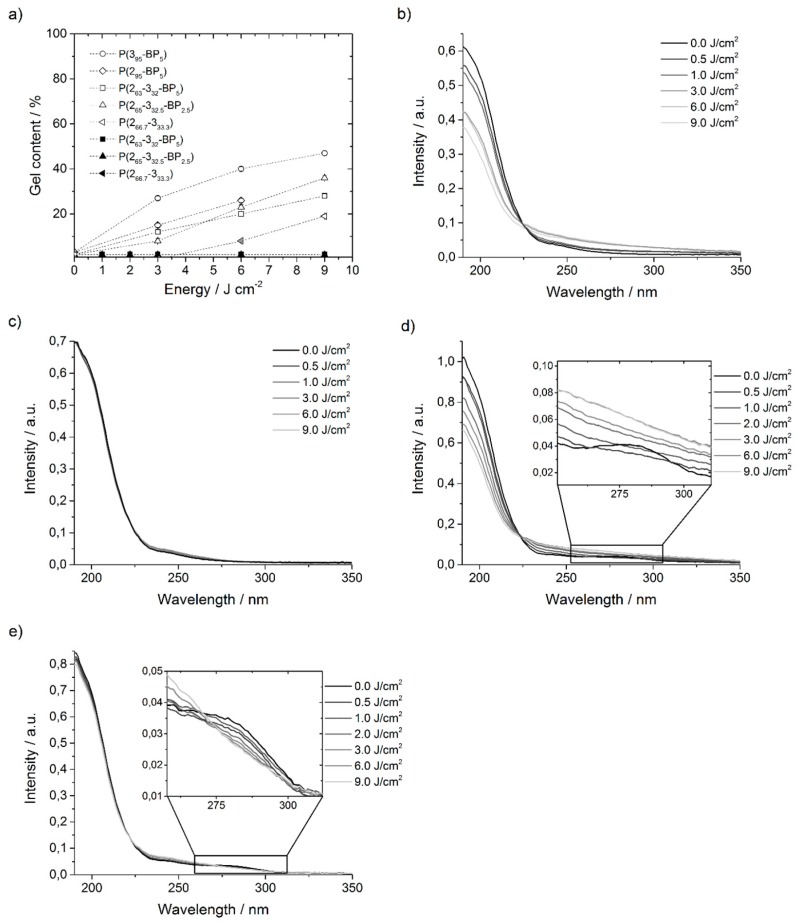
(**a**) Gel content of polymer networks consisting of different versions of **P(2-3-BP)** as a function of the irradiation energy, irradiated at λ = 254 nm (open symbols, Table S7) and λ = 365 nm (filled symbols, Table S6), **P(3_95_-BP_5_)** = (**M1**/**M2**/**M3** 5:0:95), **P(2_95_-BP_5_)** = (**M1**/**M2**/**M3** 5:95:0), **P(2_63_-3_32_-BP_5_)** = (**M1**/**M2**/**M3** 5:63:32), **P(2_65_-3_32.5_-BP_2.5_)** = (**M1**/**M2**/**M3** 2.5:65:32.5) and **P(2_66.7_-3_33.3_)** = (**M1**/**M2**/**M3** 0:66.7:33.3). (**b**–**e**) UV absorption spectra of **P(2_66.7_-3_33.3_)** films irradiated at (**b**) 254 nm and (**c**) 365 nm and of **P(2_65_-3_32.5_-BP_2.5_)** films irradiated at (**d**) 254 nm and (**e**) 365 nm. The measurements were conducted after irradiation with 0–9 J·cm^−2^.

A similar analysis was performed with the **P(2-3-BP)** polymers (Figure 1c). Polymer films were also obtained by spin-coating the respective polymers from solution onto **3EBP**-functionalized substrates as described above for the **P(4-5-BP)** samples. Again, the polymer films were cross-linked at wavelengths of 254 or 365 nm with defined energy doses. They were extracted in dichloromethane overnight to remove unattached polymer chains, and their gel content was determined. The thickness data obtained by ellipsometry for the different surface-attached **P(2-3-BP)** networks and hydrogels are listed in the supporting information (Tables S6 and S7) and summarized in Figure 4a. (As before, the subscripts of these polymers indicate the relative amount of each repeat unit in the polymer, i.e., **P(2_65_-3_32.5_-BP_2.5_)** is a polymer made from 65% **M2**, 32.5% **M3**, and 2.5% **M1**). 

In contrast to the **P(4-5-BP)** polymers, no networks formed when different **P(2-3-BP)** polymers were irradiated at λ = 365 nm, regardless of the energy dose used (up to 9 J·cm^−2^) or the amount of BP units in the polymer chain (Figure 4a and Table S6 in the Supporting Information). On the other hand, when the same experiments were repeated with higher energy photons at λ = 254 nm, gel contents of up to 47% were reached (Figure 4a). Networks made from polymer **P(2_63_-3_32_-BP_5_**) with 5% BP repeat units had a gel content that was similar to that of networks made from **P(2_65_-3_32.5_-BP_2.5_**) with 2.5% BP repeat units, i.e., the number of BP units did not significantly affect the gel content. Interestingly, polymer **P(2_66.7_-3_33.3_)** with no benzophenone groups self-crosslinked to a significant extent (19% gel content when irradiated with 9 J·cm^−2^) at 254 nm, but not when irradiated at the same dose at 365 nm (Figure 4a). To understand this, the network formation of **P(2_66.7_-3_33.3_)** was also studied by recording UV spectra of films that had been irradiated at 254 nm (Figure 4b) and 365 nm, respectively (Figure 4c), and by comparing them to UV spectra of **P(2_65_-3_32.5_-BP_2.5_)** that had been likewise treated (Figure 4d,e). The spectra of **P(2_66.7_-3_33.3_)** showed that the UV absorption bands at ~200 nm decreased from 0 to 9 J·cm^−2^ when the sample was irradiated at 254 nm (Figure 4b) and that the whole shape of the curve started to alter. Since at the same time a network formation was observed (Figure 4a, Table S7), this indicates a structural change of the polymer, which led to cross-linking. UV absorption at wavelengths around 200 nm are characteristic for an electron transition from the π-orbital to an antibonding π*-orbital of a C=C bond [28]. Since there are many double bonds in the **P(2-3**) polymer backbone, cross-linking can apparently occur also via those groups, presumably via a [2 + 2] cycloaddition involving the alkenyl group (which is not present in the corresponding poly(oxonorbornenes) that undergo chain cleavage when irradiated at 254 nm). When the same investigation was repeated with **P(2_66.7_-3_33.3_)**-films irradiated at λ = 365 nm, the UV absorption spectra did not show any decrease of the peak at 200 nm (Figure 4c), which matches the finding that no network formation was observed (Figure 4a). This verifies the assumption that cross-linking occurred also via a reaction involving the alkenyl double bonds, for which the activation energy is provided by photons at λ = 254 nm, while irradiation at 365 nm is insufficient. The same set of experiments was performed with **P(2_65_-3_32.5_-BP_2.5_)** films, which had a similar ratio of functional repeat units as **P(2_66.7_-3_33.3_)**, but additionally a small amount of BP groups. When irradiated at 365 nm, the UV absorption spectra of **P(2_65_-3_32.5_-BP_2.5_)** only show a slight decrease of the peak at 200 nm, but a steady decrease of the UV absorption band of BP at ~280 nm with increasing energy dose (Figure 4e). This indicates that the energy at λ = 365 nm was enough for the activation of the BP groups. However, thickness measurements showed that no network formed. When the **P(2_65_-3_32.5_-BP_2.5_)** films were irradiated at 254 nm, the UV absorption band of the BP group (~280 nm) vanished already at 0.5 J·cm^−2^ (Figure 4d). Again, when the sample was irradiated with higher energy doses, the whole shape of the curve started to alter. Those results are consistent with the ones of the pure **P(2_66.7_-3_33.3_)**-films irradiated at 254 nm, only the gel content and thus the degree of cross-linking was higher for **P(2_65_-3_32.5_-BP_2.5_)**. Thus, for the poly(alkenylnorbornenes) here investigated, network formation at λ = 254 nm occurs via a synergistic effect of BP-induced cross-linking and [2 + 2] cycloaddition. This finding also explains why **P(3_95_-BP_5_)** cross-linked more efficiently than **P(2_95_-BP_5_)** (Figure 4a): the fully substituted double bond of **P(2_95_-BP_5_)** is sterically more hindered than the only triply substituted one of **P(3_95_-BP_5_)**, and thus **P(2_95_-BP_5_)** has a lower propensity to undergo a [2 + 2] cycloaddition.

When the Boc protective groups of the **P(2-3-BP)** polymers were removed with hydrochloric acid to obtain surface-attached hydrogels, this treatment near-quantitatively destroyed the network (see Table S7 of the supporting information). For example, the polymer network consisting of **P(3_95_-BP_5_)** had a layer thickness of 49 nm after irradiation with 9 J·cm^−2^ and extraction, which decreased after deprotection to a thickness of only 5 nm. This suggested that in the **P(2-3-BP)** networks, most of the cross-links formed via the C–H groups in the Boc groups. To confirm this, polymer **P(2_65_-3_32.5_-BP_2.5_**) was first deprotected and then spin coated on a **3EBP**-functionalized wafer. After illumination at λ = 245 nm with 9 J·cm^−2^, a surface-attached hydrogel with a gel content of 60% was formed, while the polymer coating made from the protected version of this polymer, treated under the same conditions, yielded only a gel content of 36%. This indicated that the Boc protective groups sterically shielded the other C–H bonds of the polymer, so that BP cross-linking mainly occurred with the C–H bonds in these protective group.

In general, the ability of a polymer to effectively attach to a substrate by cross-linking depends on the exact circumstances of the network or hydrogel formation process. Körner et al. used a simple percolation model to describe the formation of polymer networks as a function of the reaction conditions and the reaction time [29]. In this model, polymers first form gel clusters inside the polymer layer, and a network is obtained once these clusters percolate through the entire polymer film. For UV irradiation of polymer films from above (e.g., using poly(styrene) containing additional BP repeat units, cross-linked at λ = 365 nm), it was observed that there was a long induction period before any macroscopic network formation was detected, as the formation of the clusters started at the air-polymer interface, and slowly percolated down to the polymer-substrate interface. In this particular case, a significant amount of network formation occurred only at an energy dose of >10 J·cm^−2^ (corresponding to 100 min irradiation time at 2.2 mW·cm^−2^) [29]. Thus, it is possible that for polymers of the **P(2-3-BP)** family, such a gel cluster formation is also very slow, so that no network percolation is observed at 365 nm in the energy range investigated. When irradiated with 254 nm, however, these polymers formed surface-attached polymer networks, albeit with a low gel content, and with synergistic BP cross-linking and double-bond-induced cross-linking. The poly(oxonorbornene) family **P(4-5-BP)**, on the other hand, degraded when irradiated at 254 nm at energy doses higher than 0.5 J·cm^−2^. Unlike **P(2-3-BP),** those polymers did not have the additional alkenyl substituent on the backbone. This double bond, however, seems to be a protection against main chain cleavage at λ = 254 nm, since it can absorb the high-energy photons via a π → π* transition. Additionally, this double bond assists network formation by its ability to self-crosslink at that wavelength. At 365 nm, on the other hand, the cross-linking behavior of the **P(4-5-BP)** copolymers was textbook-like, and with higher energy doses higher gel contents were reached. The finding that the **P(4-5-BP)** polymers undergo chain scission by photons having a wavelength of 254 nm is in line with the results of Christensen et al., who previously observed that highly abstractable hydrogens especially on the main chain tend to cause chain scission [19].

Another factor affecting the reactivity of a certain C–H bond in C–H insertion reactions, besides their electronic activation, is their steric availability. C–H abstraction can be sterically hindered by flexible, large side groups that can shield the C–H bonds near the polymer backbone [15]. For the polymers used in this study, the C–H bonds in the Boc group and the C–H bonds in the alkyl or alkenyl substituents were the sterically most easily accessible ones. In the norbornene polymers of the **P(2-3-BP)** family, the Boc groups effectively shielded the C–H bonds near the backbone, so that cross-linking via the BP units mainly occurred via the Boc group. As a result, these networks disintegrated when the Boc groups were cleaved off. Only when the Boc groups were removed before the network formation, the C–H groups nearer to the backbone were significantly involved in the cross-linking process. In case of the poly(oxanorbornenes) **P(4-5-BP)** family, on the other hand, BP could abstract hydrogen atoms from either the Boc groups or from the backbone. While the C–H bonds on the Boc group had less steric hindrance, the C–H bonds next to the oxygen and nitrogen atoms could be readily abstracted due to their lower bond energies, despite the higher steric hindrance.

## 4. Conclusions

In this work, the formation of surface-attached polymer networks and hydrogels using several poly(oxonorbornenes) and poly(alkenylnorbornenes) with benzophenone-containing repeat units was investigated in detail. The polymers were spin-coated onto benzophenone-functionalized silicon wafers and irradiated at a wavelength of 254 or 365 nm, respectively, with different energy doses. The gel content of the resulting polymer networks and hydrogels was determined by ellipsometry, and the functional groups involved in the cross-linking process were analyzed using UV spectroscopy. The results show that the cross-linking of these polymers depended strongly on the radiation wavelength and the energy does used. This could be related to subtle changes of the repeat unit structures and to steric as well as electronic effects.

Polymers belonging to the **P(2-3-BP)** family showed no network formation when irradiated at 365 nm. Irradiation with 254 nm, on the other hand, resulted in network formation due to synergistic effects between double bond cross-linking involving the alkenyl substituent on the five-membered ring of the polymer backbone on the one hand, and BP-induced cross-linking involving the Boc groups on the other hand. However, these networks were destroyed upon removal of the Boc group, as most network cross-links due to BP involved the Boc group. In case of the deprotected **P(2-3-BP)** polymers, on the other hand, hydrogels could also be formed via the other alkyl C–H bonds, which ceased to be sterically shielded after removal of the Boc groups. Thus, the presence of Boc groups leads to readily degradable polymer networks if such a feature is desired; if not, however, polymers should be deprotected prior to network formation by UV irradiation to reduce mass loss by deprotection. Apparently, the presence of the additional alkenyl substituent in the **P(2-3-BP)** polymers also protected them from main chain scission when irradiated at 254 nm.

The poly(oxonorbornenes) of the **P(4-5-BP)** family had textbook-like cross-linking behavior when irradiated at λ = 365 nm, however they were degraded at λ = 254 nm when the irradiation energy dose was higher than 0.5 J·cm^−2^. It is likely that the large number of heteroatoms in these polymers, and thus the large number of C–H bonds with lower dissociation energy, led to side reactions ultimately causing chain scission (analogous to the situation in PEO). It appears that the oxonorbornene backbone is thus not suitable for these reaction conditions. This could be investigated in future studies by comparing poly(norbornenes) and structurally identical poly(oxonorbornenes). The design rules thus obtained (avoidance of heteroatoms, beneficial effect of main-chain alkenyl substituents, avoidance of bulky, C–H rich protective groups) will help to pre-select interesting structures for the next generation of BP cross-linkable poly(norbornenes) intended for surface-attached polymer networks and hydrogels, or other kinds of UV-activated reactions.

## Figures and Tables

**Table 1:** Molar compositions, molecular weights (*M*_n_ and *M*_w_) and polydispersity indices *Ð*_M_ of the poly(alkenylnorbornenes) **P(2-3-BP)** and the poly(oxonorbornenes) **P(4-5-BP)**. *M*_n_, *M*_w_ and *Ð*_M_ were determined by gel permeation chromatography in chloroform using PMMA standards.

Polymer	Molar composition/mol %					Molecular weight/kg·mol^−1^		*Ð*_M_
	M1	M2	M3	M4	M5	*M*_n,exp_	*M*_w,exp_	
**P(4_90_-BP_10_)**	10	-	-	90	-	83	147	1.76
**P(4_85_-BP_15_)**	15	-	-	85	-	82	156	1.91
**P(2_95_-BP_5_)**	5	95	-	-	-	54	67	1.23
**P(3_95_-BP_5_)**	5	-	95	-	-	50	72	1.46
**P(4_95_-BP_5_)**	5	-	-	95	-	81	114	1.41
**P(4_95_-BP_5_)**	5	-	-	95	-	81	153	1.87
**P(4_95_-BP_5_)**	5	-	-	95	-	82	384	4.69
**P(2_63_-3_32_-BP_5_)**	5	63	32	-	-	45	51	1.14
**P(4_85_-5_10_-BP_5_)**	5	-	-	85	10	84	139	1.65
**P(4_85_-5_10_-BP_5_)**	5	-	-	85	10	88	480	5.48
**P(2_65_-3_32.5_-BP_2.5_)**	2.5	65	32.5	-	-	53	71	1.33
**P(2_66.7_-3_33.3_)**	-	66.7	33.3	-	-	24	59	2.50

## Supplementary Materials

The following are available online at www.mdpi.com/2073-4360/9/12/686/s1.
