# Supplementary material for: On the Limits of Benzophenone as Cross-Linker for Surface-Attached Polymer Hydrogels
Source: Polymers (Basel). 2017 Dec 7;9(12):686. doi: 10.3390/polym9120686 (PMC6418956; doi:10.3390/polym9120686)
Supplement: Supplementary file 1 [file polymers-09-00686-s001.pdf]

Supporting Information

on the Limits of Benzophenone as Cross-Linker in Polymer Chemistry

Esther K. Riga,[#] Julia S. Saar,[#] Roman Erath, Michelle Hechenbichler, and Karen Lienkamp*

E. K. Riga, J. S. Saar, R. Erath, M. Hechenbichler, Dr. K. Lienkamp, Freiburg Center für Interactive Materials and Bioinspired Technologies (FIT) and Department of Microsystems Engineering (IMTEK), Albert-Ludwigs-Universität, Georges-Köhler-Allee 105, 79110 Freiburg, Germany; E-Mail: lienkamp@imtek.uni-freiburg.de

[#] contributed equally

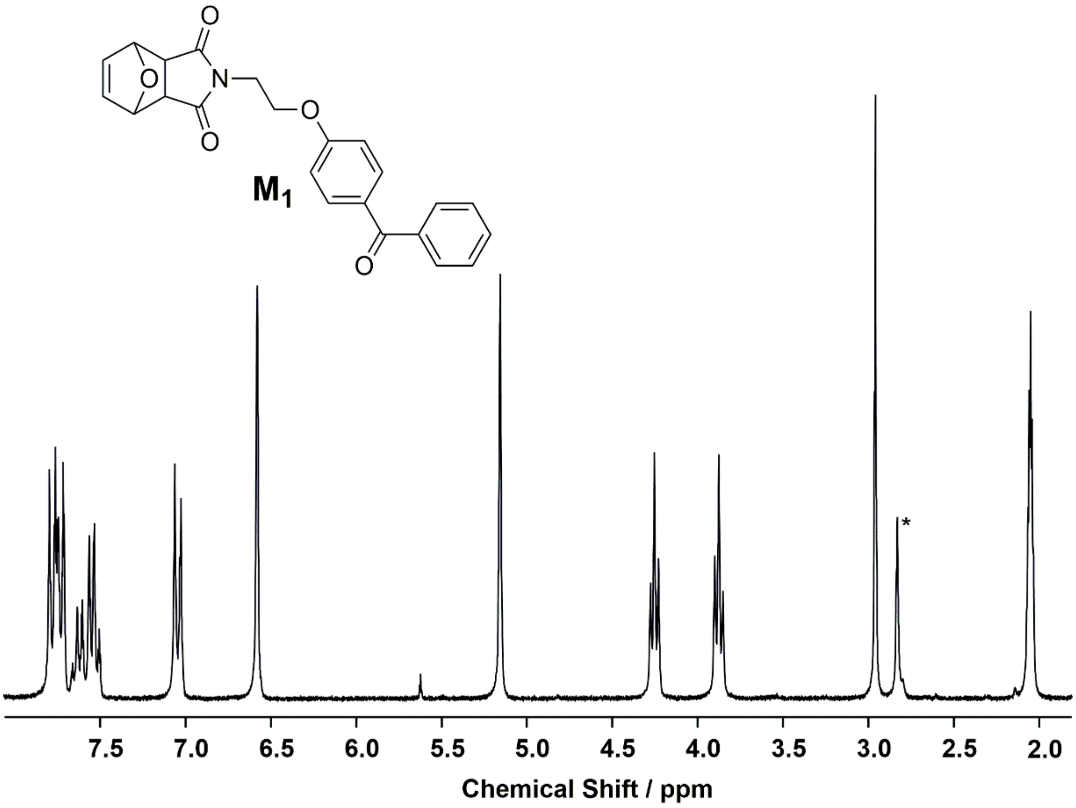

Figure S1: ^{1}H NMR spectra of the benzophenone-containing monomer **M1**. The water peak of the solvent is marked with an asterisk (*).

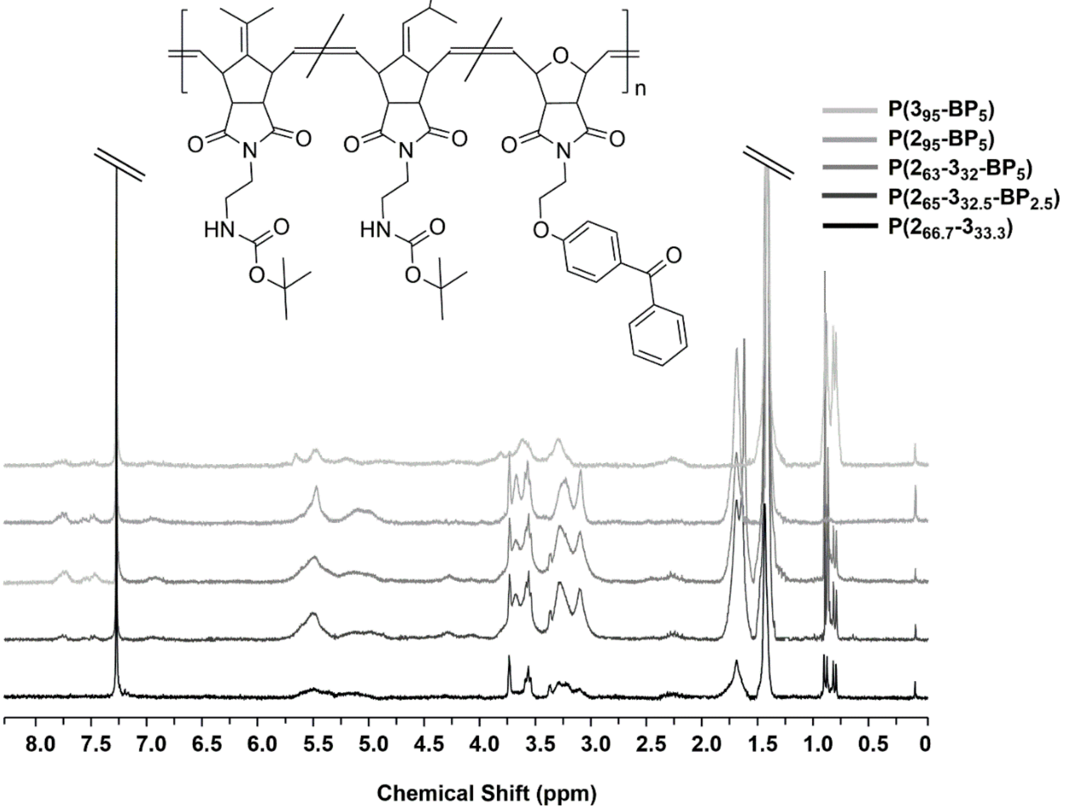

Figure S2: ^{1}H NMR spectra of the protected copolymers **P(2-3)-BP**.

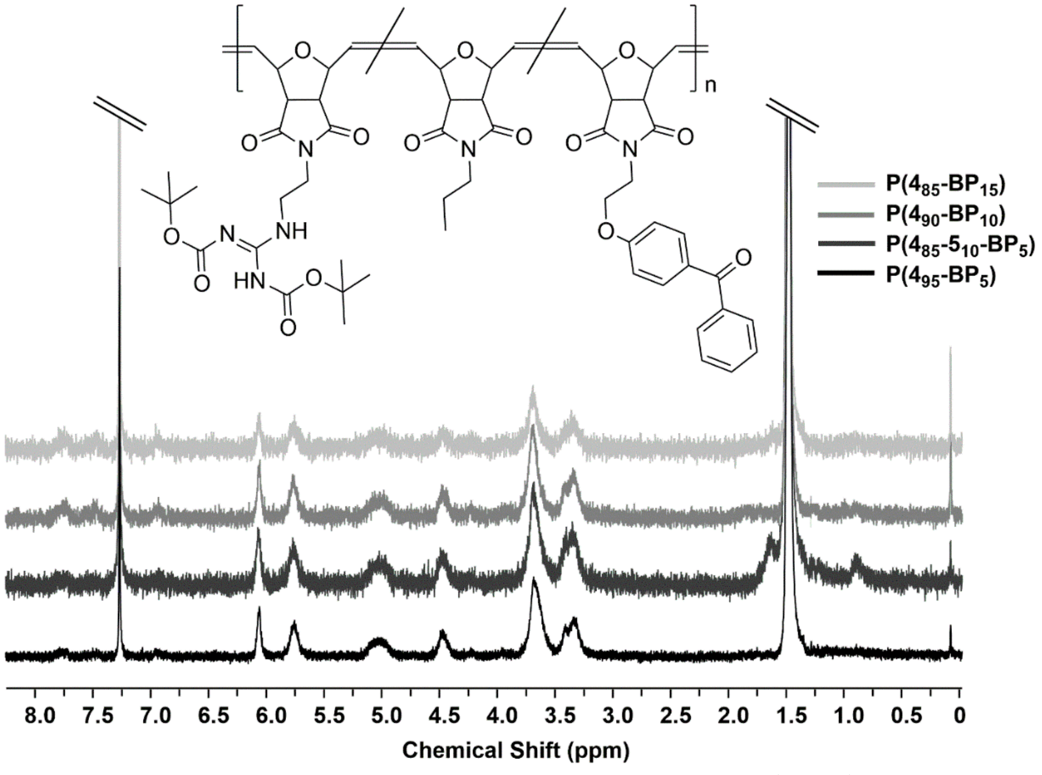

Figure S3: ^{1}H NMR spectra of the protected copolymers **P(4-5-BP)**.

The GPC data is given in Table S1 and S2, the elugrams are given in Figure S4 and S5.

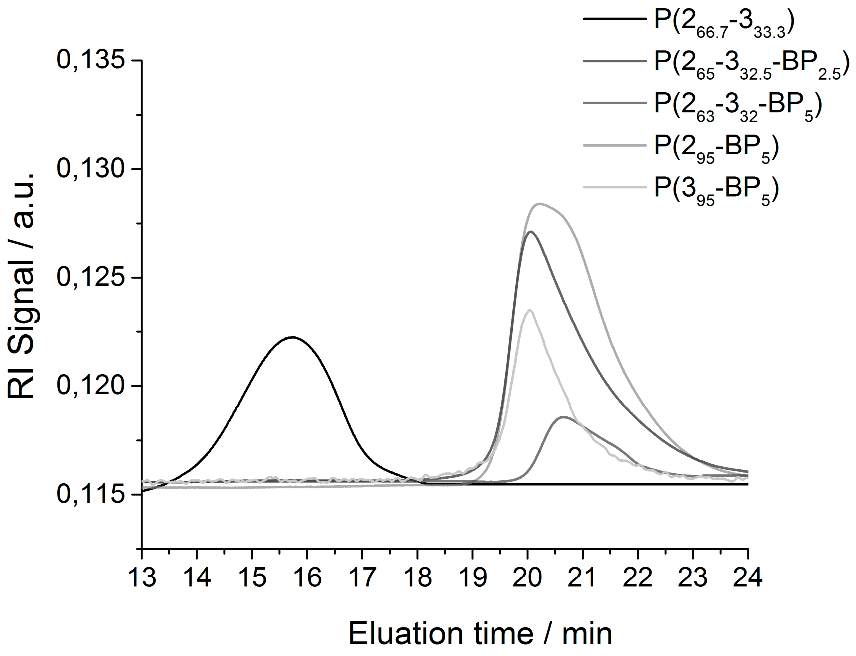

Figure S4: GPC elugrams of the copolymers **P(2-3-BP)**.

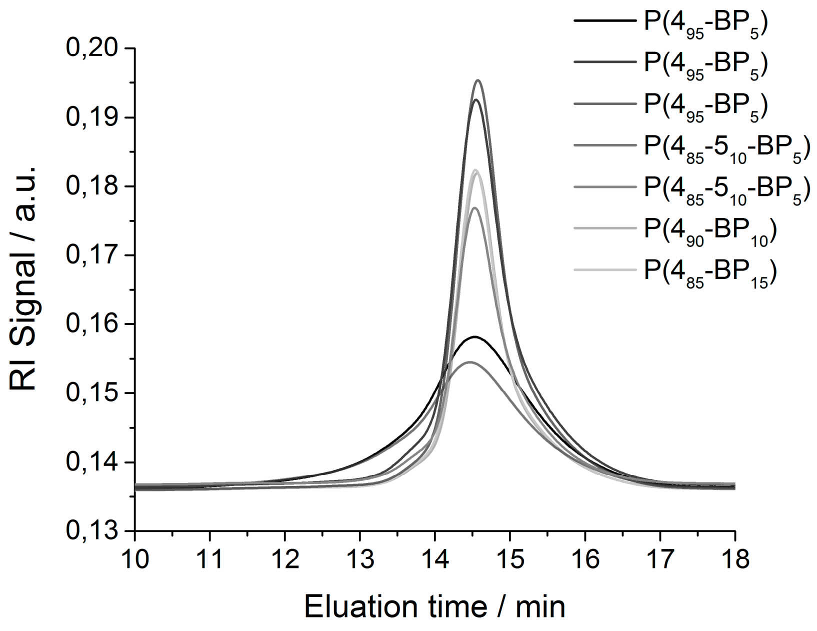

Figure S5: GPC elugrams of the copolymers **P(4-5-BP)**.

Table S1: The scheduled quantity of the ring-opening metatheses polymerization of the norbornene imide monomers M1, M2 and M3.

amount of substance / mmol				initial weight / mg				yield / mg	yield / %	molecular weight [a]/ kg mol^{-1}		$Đ_M$
M1	M2	M3	G3	M1	M2	M3	G3			$M_{n,ex}$	$M_{w,exp}$	
0.029	-	0.555	0.002	11.4	-	200	1.57	122	42	50	72	1.46
0.110	0.220	-	0.001	3.43	75.0	-	0.580	21.0	18	54	67	1.23
0.034	0.434	0.216	0.003	13.4	151	78.4	1.80	81.0	26	45	51	1.14
0.022	0.577	0.289	0.003	8.90	201	104	2.27	313	67	53	71	1.33
-	0.400	0.200	0.002	-	140	72.8	1.55	60	42	24	59	2.50

[a] Determined using GPC in chloroform relative to PMMA standards.

Table S2: The scheduled quantity of the ring-opening metatheses polymerization of the oxonorbornene imide monomers M1, M4 and M5.

amount of substance / mmol				initial weight / mg				yield / %	molecular weight [a]/ kg mol^{-1}		$Đ_M$
M1	M4	M5	G3	M1	M4	M5	G3		$M_{n,ex}$	$M_{w,exp}$	
0.03	0.57	-	0.003	11.7	257	-	1.95	~90	81	114	1.41
0.03	0.57	-	0.003	11.7	257	-	1.95	~90	81	153	1.87
0.03	0.57	-	0.003	11.7	257	-	1.95	~90	82	384	4.69
0.03	0.51	0.06	0.003	11.7	230	12.43	1.84	~90	84	139	1.65
0.03	0.51	0.06	0.003	11.7	230	12.43	1.84	~90	88	480	5.48
0.06	0.54	-	0.003	23.3	243	-	1.95	~90	83	147	1.76
0.09	0.51	-	0.003	35.0	230	-	1.95	~90	82	156	1.91

[a] Determined using GPC in chloroform relative to PMMA standards.

Table S3: Layer thickness and gel content of polymer coatings containing M1 in relation to cross-linking energies and monomer ratios.

[M1]:[M4]: [M5]	$M_{n,exp}$ [a] / kg mol^{-1}	E [b] / J cm^{-2}	d_0 [c] / nm	$d_{extract}$ [d] / nm	gel content [e] / %
5:95:0	82	0.2	280 ± 4	167 ± 1	60 ± 1
5:95:0	82	0.5	280 ± 2	211 ± 3	75 ± 1
5:95:0	82	1	289 ± 7	198 ± 4	69 ± 3
5:95:0	82	2	289 ± 9	162 ± 10	56 ± 5
5:95:0	82	3	270 ± 1	117 ± 5	43 ± 1
5:95:0	82	4	271 ± 7	59 ± 1	22 ± 1
5:95:0	82	6	269 ± 3	20 ± 3	8 ± 1
5:85:10	88	0.2	294 ± 3	178 ± 1	60 ± 1
5:85:10	88	0.5	286 ± 4	216 ± 3	75 ± 2
5:85:10	88	1	277 ± 13	201 ± 9	73 ± 1
5:85:10	88	2	278 ± 2	167 ± 9	60 ± 3
5:85:10	88	3	280 ± 2	106 ± 5	37 ± 2
5:85:10	88	4	275 ± 5	54 ± 11	20 ± 4
5:85:10	88	6	303 ± 16	14 ± 12	4 ± 4

[a] Determined using GPC in chloroform relative to PMMA standards. [b] Energy dose of radiation: 254 nm UV radiation. [c] Layer thickness of the sample bevor extraction, determined using ellipsometry. [d] Layer thickness of the sample after extraction, determined using ellipsometry. [e] Gel content was calculated using ($d_{extract}/d_0$*100).

Table S4: Layer thickness and gel content of polymer coatings containing M1 in relation to cross-linking energies and monomer ratios.

[M1]:[M4]: [M5]	$M_{n,exp}$ [a] / kg mol^{-1}	E [b] / J cm^{-2}	d_0 [c] / nm	$d_{extract}$ [d] / nm	gel content [e] / %
5:95:0	81	1	308 ± 6	1 ± 0	0 ± 0
5:95:0	81	1.5	309 ± 4	20 ± 2	6 ± 1
5:95:0	81	2	316 ± 4	80 ± 12	24 ± 4
5:95:0	81	3	303 ± 6	160 ± 3	53 ± 2
5:95:0	81	4	309 ± 2	189 ± 5	61 ± 1
5:95:0	81	5	307 ± 2	218 ± 3	71 ± 1
5:95:0	81	6	309 ± 5	238 ± 1	77 ± 1
5:95:0	81	7	270 ± 5	201 ± 3	74 ± 1
5:95:0	81	8	277 ± 2	210 ± 4	76 ± 1
5:95:0	81	9	290 ± 13	221 ± 5	76 ± 2
5:95:0	81	10	287 ± 18	219 ± 8	77 ± 4
10:90:0	83	1	274 ± 7	150 ± 4	54 ± 1
10:90:0	83	1.5	307 ± 45	214 ± 23	70 ± 3
10:90:0	83	2	268 ± 17	198 ± 10	74 ± 1
10:90:0	83	3	262 ± 2	219 ± 5	84 ± 2
10:90:0	83	4	262 ± 5	229 ± 6	87 ± 1
10:90:0	83	5	271 ± 7	237 ± 3	87 ± 1
10:90:0	83	6	268 ± 14	233 ± 11	87 ± 2
15:85:0	82	1	304 ± 13	233 ± 6	77 ± 1
15:85:0	82	1.5	310 ± 3	256 ± 7	83 ± 2
15:85:0	82	2	300 ± 5	267 ± 5	89 ± 2
15:85:0	82	3	310 ± 5	273 ± 3	88 ± 2
15:85:0	82	4	313 ± 6	280 ± 4	90 ± 1
15:85:0	82	5	300 ± 3	275 ± 1	91 ± 1
15:85:0	82	6	309 ± 4	278 ± 6	90 ± 1

[a] Determined using GPC in chloroform relative to PMMA standards. [b] Energy dose of radiation: 365 nm UV radiation. [c] Layer thickness of the sample bevor extraction, determined using ellipsometry. [d] Layer thickness of the sample after extraction, determined using ellipsometry. [e] Gel content was calculated using ($d_{extract}/d_0$*100).

Table S5: Layer thickness and gel content of polymer coatings containing M1 in relation to cross-linking energies and monomer ratios.

[M1]:[M4]: [M5]	$M_{n,exp}$ [a] / kg mol^{-1}	E [b] / J cm^{-2}	d_0 [c] / nm	$d_{extract}$ [d] / nm	gel content [e] / %	$d_{deprotect}$ [f] / nm	$d_{deprotect}$ [d] - $d_{extract}$ [f] / nm
5:95:0	82	0.2	294 ± 4	1 ± 0	0 ± 0	2 ± 0	1 ± 0
5:95:0	82	0.5	286 ± 6	1 ± 0	0 ± 0	2 ± 0	1 ± 0
5:95:0	82	1	286 ± 4	3 ± 1	1 ± 0	2 ± 0	-1 ± 1

5:95:0	82	2	281 ± 8	99 ± 6	35 ± 3	37 ± 5	-62 ± 11
5:95:0	82	3	279 ± 12	148 ± 9	54 ± 2	68 ± 4	-80 ± 13
5:95:0	82	4	274 ± 6	165 ± 17	60 ± 5	76 ± 11	-89 ± 28
5:95:0	82	6	282 ± 3	203 ± 6	72 ± 2	108 ± 6	-95 ± 12
5:95:0	81	7	293 ± 6	222 ± 2	76 ± 1	110 ± 3	-122 ± 5
5:95:0	81	8	278 ± 14	212 ± 12	76 ±1	105 ± 10	-107 ± 22
5:95:0	81	9	286 ± 8	219 ± 9	76 ± 1	109 ± 8	-110 ± 17
5:95:0	81	10	279 ± 4	229 ± 3	82 ± 1	113 ± 6	-116 ± 9
5:85:10	88	0.2	266 ± 6	1 ± 0	0 ± 0	2 ± 0	1 ± 0
5:85:10	88	0.5	279 ± 12	1 ± 0	0 ± 0	2 ± 0	1 ± 0
5:85:10	88	1	283 ± 2	38 ± 15	14 ± 5	12 ± 9	-26 ± 24
5:85:10	88	2	268 ± 1	143 ± 12	53 ± 4	67 ± 5	-76 ± 17
5:85:10	88	3	270 ± 4	174 ± 4	65 ± 1	87 ± 3	-87 ± 7
5:85:10	88	4	291 ± 14	201 ± 7	69 ± 5	102 ± 3	-99 ± 10
5:85:10	88	6	274 ± 4	206 ± 3	75 ± 2	113 ± 3	-93 ± 6
5:85:10	84	7	260 ± 7	204 ± 6	79 ± 1	111 ± 2	-93 ± 8
5:85:10	84	8	258 ± 2	209 ± 3	81 ± 1	109 ± 6	-100 ± 9
5:85:10	84	9	250 ± 2	203 ± 3	81 ± 0	106 ± 6	-97 ± 9
5:85:10	84	10	250 ± 2	206 ± 3	82 ± 1	116 ± 6	-90 ± 9

[a] Determined using GPC in chloroform relative to PMMA standards. [b] Energy dose of radiation: 365 nm UV radiation. [c] Layer thickness of the sample bevor extraction, determined using ellipsometry. [d] Layer thickness of the sample after extraction, determined using ellipsometry. [e] Gel content was calculated using ($d_{extract}/d_0*100$). [f] Layer thickness of the sample after deprotection, determined using ellipsometry.

Table S6: Layer thickness and gel content of polymer coatings containing M1 in relation to cross-linking energies and monomer ratios.

[M1]:[M2]: [M3]	$M_{n,exp}$ [a] / kg mol^{-1}	E [b] / J cm^{-2}	d_0 [c] / nm	$d_{extract}$ [d] / nm	gel content [e] / %
5:63:32	45	0	139 ± 1	4 ± 3	2 ± 2
5:63:32	45	1	140 ± 1	3 ± 2	2 ± 1
5:63:32	45	2	139 ± 1	3 ± 2	2 ± 1
5:63:32	45	3	127 ± 1	3 ± 2	2 ± 1
5:63:32	45	6	140 ± 1	3 ± 2	2 ± 1
5:63:32	45	9	139 ± 1	3 ± 2	2 ± 1
2.5:65:32.5	53	0	128 ± 1	3 ± 2	2 ± 1
2.5:65:32.5	53	1	139 ± 1	3 ± 2	2 ± 1
2.5:65:32.5	53	2	139 ± 1	4 ± 2	2 ± 1
2.5:65:32.5	53	3	127 ± 1	4 ± 2	2 ± 1
2.5:65:32.5	53	6	139 ± 1	3 ± 2	2 ± 1
2.5:65:32.5	53	9	139 ± 1	3 ± 2	2 ± 1
0:66.7:33.3	24	0	170 ± 1	2 ± 2	2 ± 2
0:66.7:33.3	24	1	169 ± 1	2 ± 2	2 ± 2
0:66.7:33.3	24	2	171 ± 1	3 ± 2	2 ± 2
0:66.7:33.3	24	3	170 ± 1	3 ± 2	2 ± 2

0:66.7:33.3	24	6	170 ± 1	2 ± 2	2 ± 2
0:66.7:33.3	24	9	169 ± 1	3 ± 2	2 ± 2

[a] Determined using GPC in chloroform relative to PMMA standards. [b] Energy dose of radiation: 365 nm UV radiation. [c] Layer thickness of the sample bevor extraction, determined using ellipsometry. [d] Layer thickness of the sample after extraction, determined using ellipsometry. [e] Gel content was calculated using ($d_{extract}/d_0*100$).

Table S7: Layer thickness and gel content of polymer coatings containing M1 in relation to cross-linking energies and monomer ratios.

[M1]:[M2]: [M3]	$M_{n,exp}$ [a] / kg·mol^{-1}	E [b] / J cm^{-2}	d_0 [c] / nm	$d_{extract}$ [d] / nm	gel content [e] / %	$d_{deprotect}$ [f] / nm
5:95:0	50	0	107 ± 2	3 ± 2	3 ± 2	-
5:95:0	50	3	107 ± 2	16 ± 1	15 ± 1	3 ± 2
5:95:0	50	6	106 ± 2	28 ± 1	26 ± 1	3 ± 2
5:0:95	54	0	105 ± 1	3 ± 2	3 ± 2	-
5:0:95	54	3	105 ± 1	28 ± 1	27 ± 1	4 ± 2
5:0:95	54	6	104 ± 1	42 ± 1	40 ± 1	5 ± 2
5:0:95	54	9	105 ± 1	49 ± 1	47 ± 1	5 ± 2
5:63:32	45	0	93 ± 1	2 ± 2	2 ± 2	-
5:63:32	45	3	94 ± 1	11 ± 1	12 ± 1	3 ± 2
5:63:32	45	6	95 ± 1	19 ± 1	20 ± 1	3 ± 2
5:63:32	45	9	94 ± 1	26 ± 1	28 ± 1	2 ± 2
2.5:65:32.5	53	0	124 ± 1	3 ± 2	2 ± 2	-
2.5:65:32.5	53	3	124 ± 1	10 ± 1	8 ± 1	4 ± 2
2.5:65:32.5	53	6	123 ± 1	28 ± 1	23 ± 1	4 ± 2
2.5:65:32.5	53	9	124 ± 1	45 ± 1	36 ± 1	5 ± 2
2.5:65:32.5 [g]	53	9	45 ± 1	27 ± 1	60 ± 1	-
0:66.7:33.3	24	0	170 ± 1	2 ± 2	2 ± 2	-
0:66.7:33.3	24	0.5	169 ± 1	2 ± 2	1 ± 2	-
0:66.7:33.3	24	1	171 ± 1	2 ± 2	1 ± 2	-
0:66.7:33.3	24	3	170 ± 1	3 ± 2	1 ± 2	-
0:66.7:33.3	24	6	170 ± 1	13 ± 1	8 ± 1	6 ± 2
0:66.7:33.3	24	9	169 ± 1	33 ± 2	19 ± 1	8 ± 2

[a] Determined using GPC in chloroform relative to PMMA standards. [b] Energy dose of radiation: 254 nm UV radiation. [c] Layer thickness of the sample bevor extraction, determined using ellipsometry. [d] Layer thickness of the sample after extraction, determined using ellipsometry. [e] Gel content was calculated using ($d_{extract}/d_0*100$). [f] Layer thickness of the sample after deprotection, determined using ellipsometry. [g] Polymer coatings using the deprotected polymer.
